# Water strider females use individual experience to adjust jumping behaviour to their weight within physical constraints of water surface tension

**DOI:** 10.1038/s41598-020-75564-x

**Published:** 2020-10-29

**Authors:** Minjung Baek, Katherine M. Lawin, Christina J. Codden, Hangkyo Lim, Eunjin Yang, Ho-Young Kim, Sang-im Lee, Piotr G. Jablonski

**Affiliations:** 1 Laboratory of Behavioral Ecology and Evolution, School of Biological Sciences, Seoul National University, Seoul, 08826 Korea; 2 Ecology and Evolutionary Biology (EEB), University of Arizona, Tucson, AZ 85721 USA; 3 University of St. Thomas, 2115 Summit Ave., St. Paul, MN 55105 USA; 4 Northeastern University, 360 Huntington Avenue, Boston, MA 02215 USA; 5 Notre Dame of Maryland University, 4701 North Charles St, Baltimore, MD 21210 USA; 6 Department of Mechanical and Aerospace Engineering, Seoul National University, Seoul, 08826 Korea; 7 Institute of Advanced Machines and Design, Gwanak-ro 1, Gwanak-gu, Seoul, 08826 Korea; 8 Laboratory of Integrative Animal Ecology (IAE), Department of New Biology, Daegu-Gyeongbuk Institute of Science and Technology, Daegu, 42988 Korea; 9 Museum and Institute of Zoology PAS, Wilcza 64, 00-679 Warsaw, Poland

**Keywords:** Behavioural ecology, Evolutionary ecology, Animal behaviour, Biomechanics, Entomology

## Abstract

Different species of water striders match leg speeds to their body sizes to maximize their jump take off velocity without breaking the water surface, which might have aided evolution of leg structures optimized for exploitation of the water surface tension. It is not understood how water striders achieve this match. Can individuals modify their leg movements based on their body mass and locomotor experience? Here we tested if water striders, *Gerris latiabdominis*, adjust jumping behaviour based on their personal experience and how an experimentally added body weight affects this process. Females, but not males, modified their jumping behaviour in weight-dependent manner, but only when they experienced frequent jumping. They did so within the environmental constraint set by the physics of water surface tension. Females’ ability to adjust jumping may represent their adaptation to frequent increases or decreases of the weight that they support as mating bouts, during which males ride on top of females, start or end, respectively. This suggests that natural selection for optimized biomechanics combined with sexual selection for mating adaptations shapes this ability to optimally exploit water surface tension, which might have aided adaptive radiation of Gerromorpha into a diversity of semiaquatic niches.

## Introduction

**Figure 1:**
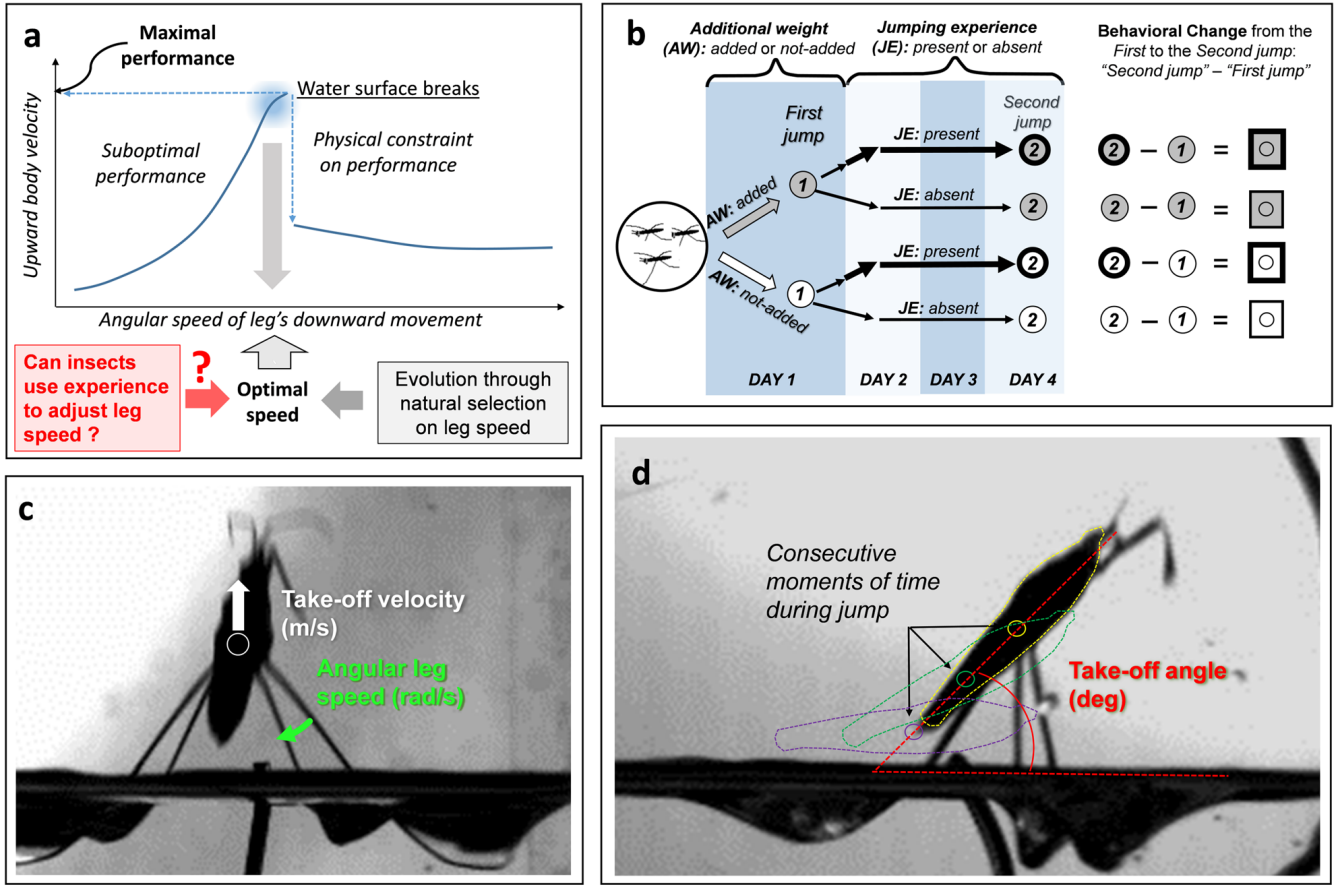
Research question and methods: the theoretical significance of determining if water striders use their personal experience to adjust their leg speed during jumping on the water surface (**a**), and the experimental scheme used in the study (**b**). In (**a**) According to our recent theoretical model (3), as the angular leg speed increases so is the jumping speed (while the time to take-off decreases; not shown here), until the moment when water surface breaks and jumping performance abruptly decreases. The optimal leg speed is just below that critical value. Water striders are able to maintain, on average, the optimal leg speed, and determining if they are able to modify their leg movements based on personal experience is the first step to evaluate if individual adjustments may be responsible for this optimal behaviour. In (**b**) on DAY 1, individual water strider of *Gerris latiabdominis* was randomly assigned to two *Additional Weight* (*AW*) treatments: *weight-added* (gray box) or *weight-not-added* (white box) treatment. *First jump* was filmed about two hours after weight addition on DAY 1. Change in jumping performance was expressed as the difference between *First jump* and *Second jump* (value at *Second jump* minus value at *First jump*) filmed three days later after some individuals had an opportunity to experience frequent jumping (*Jumping Experience* [*JE*]*-present* condition; box plots with thick lines), while others did not (*JE-absent* condition; box plots with thin lines). (**c**) and (**d**) contain graphical conceptualizations of the three response variables: take off velocity (**c**), angular leg speed (**c**) and take-off angle (**d**).

Surface-tension-dominated locomotion of water striders has attracted the attention of researchers at the interface of biology and engineering^1–3^, who are interested in designing biomimetic devices that move on the water surface. This multidisciplinary research has indicated that escape efficiency of jumps by the water striders should depend on the ability to adjust their leg movements to jumping conditions^3^. In order to maximize body velocity during jumping and to minimize latency till leaving the water surface, a jumping water strider should push the water surface downward with its long midlegs^4^ at the speed that is high enough to produce fast take-off but not higher than the upper threshold value, above which the legs break the water surface and the take-off velocity is dramatically reduced^3^ (Fig. 1a). Theoretically, this optimal leg speed depends on the body mass and on the leg length of a water strider, which vary by species^3,4^. Empirical data confirmed that different species use angular downward speeds of legs that are close to these theoretical optima calculated for each species from the species-specific body mass and leg length^3^, which assure that legs interact with un-broken surface of water. This ability might have aided^5^ adaptive evolution of leg morphology and micro-structures for optimized exploitation of water surface tension, which is associated with evolutionary colonization of water surface habitats by water striders^4,6^. It is not known how water striders achieve this optimal angular leg speed that matches the theoretical predictions and assures that legs interact with unbroken surface of water. It is possible that natural selection produces a fixed action pattern of the species-specific mid-leg movements that, on average, maximizes jumping performance for the species’ body mass and leg length. However, it is also known that many organisms, including insects, adjust their behaviour to changing environmental conditions through developmental or behavioural phenotypic plasticity^7–16^. Therefore, it is possible that individual water striders adjust their leg speed via personal experience through frequent jumping, which may result in the species-specific angular leg speed that are near the predicted optimum^3^. Therefore, we asked if water strider use experience to adjust their leg speed during locomotion (Fig. 1a).

The degree of behavioural plasticity in response to personal experience may vary not only among individuals or among species^17–19^, but also between sexes within a species^20^ if males and females face different selective pressures. Sexually selected behaviours may expose males and females to different experience, and this may result in the sex difference in the ability to adjust to environmental conditions. Water striders are a good study system to explore this issue in the context of locomotory behaviour because female water striders experience dramatic weight change while mating on the water surface. A male water strider mounts a female during mating and remains mounted for a long period, which may last for hours or even days, and females experience multiple mating bouts during their life^21–32^. Therefore, females, but not males, repeatedly experience periods of additional weight followed by periods of normal weight, and even when mating are able to perform jumps on the water surface either in response to predators or in attempts to shrug off the mating males. The frequent changes in weight are associated with changes in female’s predation risk^24–26^, and may pose a selective pressure for the female-specific abilities to adjust locomotion on the water surface to changes in the weight that is supported by the legs.

In this study, we conducted experiments (Fig. 1b) to determine whether male and female water striders *Gerris latiabdominis* use their experience to modify their leg movements during their jumps in the manner that depends on the body weight supported by their legs. We predicted that this behavioural change may be more pronounced in females, who naturally experience frequent changes in the weight that is supported by the legs. We also predicted that behavioural adjustments of jumping should be performed within the upper limits set by the physical properties of water surface.

## Results

**Figure 2:**
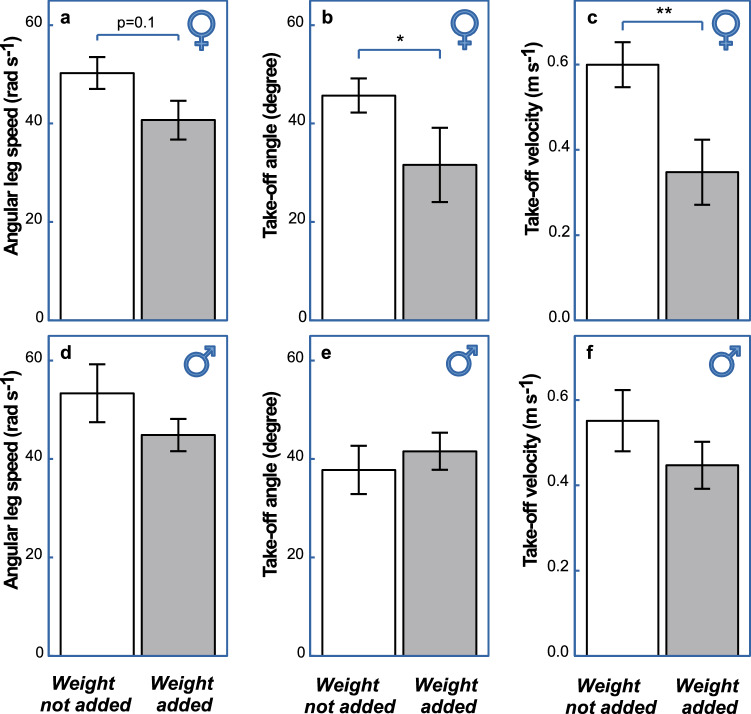
(From Supplementary Fig. 1). The effect of additional weight on jumping performance of *Gerris latiabdominis* females (**a**–**c**) and males (**d**–**f**) in *First Jumps*. Average *Angular leg speed* (**a**, **d**), *Take-off angle* (**b**, **e**), and *Take-off velocity* (**c**, **f**) of *weight-not-added* (white bars) and *weight-added* females (gray bars) are compared. Results from the Wilcoxon rank sum test (Mann–Whitney test; see Supplementary Materials Part 1 for details) are graphically presented in the figure: *P < 0.05, **P < 0.001. One marginally non-significant difference at *P* = 0.104 is marked in (**a**) to accommodate the reasoning in the text. Parametric ANOVA for this data is presented in Table 1. Additional statistical analyses are provided in Supplementary Materials Part 1. Sample sizes in **a**, **b**, **c** are 20 and 11 for *weight-not-added* and *weight-added* females respectively. Sample sizes in **d**, **e**, **f** are 15 and 16 for *weight-not-added* and *weight-added* males respectively. Error bars indicate standard errors.

*Immediate effect of additional weight on jumping performance—*We tested water striders in two conditions of the *Additional weight* treatment (Fig. 1b; names of explanatory and response variables are in bold italic and their levels/values are in italic): *weight-added* (about 50% of body mass) and *weight-not-added*. Three hours after the weight addition (DAY 1 in Fig. 1b; see Methods), we recorded three variables of water striders’ jumping behaviour and performance during a jump (*First jump*; Fig. 1b): *Angular leg speed* (Fig. 1c; rad/s; detailed explanations for the variables are in the Methods section) of downward mid-leg movement, *Take-off angle* (Fig. 1d; deg; angle between body center trajectory and water surface at take-off; body center trajectory typically follows a near straight line) and vertical *Take-off velocity* (Fig. 2c; m/s). Regardless of sex (non-significant *Sex*:*Additional weight* interaction terms in Table 1), *Angular leg speed* downward against the water surface (Fig. 2a, d) and the body’s upward vertical *Take-off velocity* (Fig. 2c, f) were smaller in *weight-added* individuals (*P* < 0.04; Table 1). In the tests with females (Fig. 2b,) the *weight-added* individuals jumped at less steep angles than the *weight-not-added* individuals did (*Take-off angle*, Wilcoxon test: *W* = 162,* P* = 0.038; Fig. 2b). No such a difference was observed for males (*W* = 109, *P* = 0.682; Fig. 2e). This difference between sexes is reflected in marginally non-significant (*p* = 0.064) interaction *Sex*:*Additional weight* (Table 1).

**Table 1:** ANOVA table for *First jump* presented in Fig. 2. Effect of *Sex* and *Additional weight* (AW) on (a) *Angular leg speed*, (b) *Take-off angle* and (c) *Take-off velocity*.

Dependent variable	Independent variable	Df	Sum Sq	Mean Sq	F value	Pr
(a) Angular leg speed	Sex	1	68	68.3	0.255	0.6154
	**Additional weight**	1	1201	1201.0	4.486	**0.0385**
	Sex:weight	1	4	4.4	0.016	0.8987
	Residuals	58	15,528	267.7		
(b) Takeoff angle	Sex	1	14	14.22	0.043	0.8372
	Additional weight	1	335	334.6	1.004	0.3205
	***Sex:weight***	1	1189	1188.8	3.567	***0.0639***
	Residuals	58	19,330	333.3		
(c) Takeoff velocity	Sex	1	0.002	0.0025	0.041	0.8397
	**Additional weight**	1	0.454	0.4542	7.508	**0.0082**
	Sex:weight	1	0.081	0.0807	1.334	0.2529
	Residuals	58	3.509	0.0605		

Significant effects are marked with bold font. Marginally significant interaction effect *Sex*:*Additional Weight* is marked in italic bold. Additional statistical analyses are shown in Supplementary Materials PART 2.

**Figure 3:**
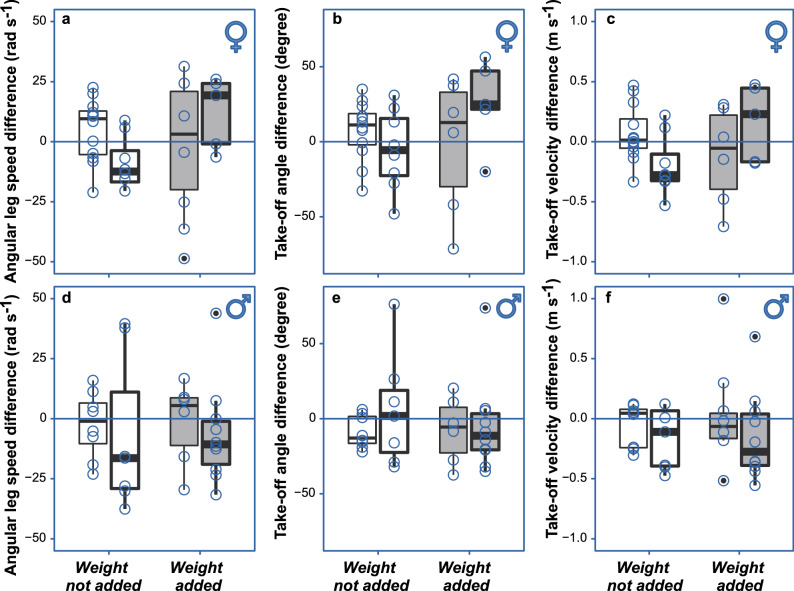
Females, but not males, modified jump performance after experiencing frequent jumps. Change in jumping performance (difference between *Second* and *First jumps*) was measured for three variables: *Angular leg speed* (**a**, **d**), *Take-off angle* (**b**, **e**) and *Take-off velocity* (**c**, **f**) in females (**a**–**c**) and males (**d**–**f**) of *G. latiabdominis*. Jumping performance of those Individuals who had an opportunity to experience frequent jumping between *First* and *Second jumps* (*Jumping Experience* [*JE*]*-present*) is represented with box plots with thick lines, and that of the individuals who did not experience frequent jumping (*JE-absent*) is represented with box plots with thin lines. Experimental scheme is explained in Fig. 1. Medians (horizontal line), upper and lower quartiles (lower and upper box margin) and [quartile + 1.5 interquartile range] values (vertical line) are shown. Gray and white boxes indicate *weight-added* and *weight-not-added* groups of the *Additional weight* treatment respectively. Thin and bold lines indicate *JE-absent* and *JE-present* conditions, respectively. Statistical results are in Table 1. Sample sizes in a-c were 12, 8, 6, and 5 from left to right in each panel. Sample sizes in d-f were 8, 7, 6, and 10 from left to right.

*Effect of experience on the adjustments of jumping behaviour*—In order to address the main question of our study and to test the effect of individual’s experience on the adjustments of jumping behaviour, we subjected males and females in the *weight-not-added* and *weight-added* groups to two conditions of *Jumping experience* (*JE*) treatment (Fig. 1b): *presence* and *absence* of frequent jumping over three consecutive days (DAY 2, DAY 3, and DAY 4 in Fig. 1b) between the recordings of the *First* (reported above) and *Second jumps* (Fig. 1b). For each of the three variables, we used the difference between the *Second* and *First jumps* (Fig. 1b) as the measure of behavioural change during the three days. The results indicate that jumping experience facilitated weight-dependent behavioural changes in females (Fig. 3; Table 2), especially for *Angular leg speed* and *Take-off velocity* (notice significant interaction terms in Table 2a, c). *Weight-added* females (gray-filled boxes in Fig. 3) changed their performance between *First* and *Second jumps* differently than the *weight-not-added* females by increasing the speed of their leg movements (Fig. 3a; Table 2a) and increasing their vertical take-off velocity (Fig. 3c; Table 2c) after experiencing frequent jumps (i.e. in *JE-present* condition, marked by bold lines in Fig. 3, in comparison to *JE-absent* condition, marked with thin lines in Fig. 3), while *weight-not-added* females modified their behaviour in the opposite manner (Fig. 3a–c; white filled boxes). There were no such significant changes in males (Fig. 3d–f; Table 2). An additional analysis of only *Second jumps* for each sex revealed no differences among the treatment groups, and no interaction effects (in Supplementary Materials PART 3), which indicates that the weight-specific behavioural adjustments by female water striders compensated for the initial effect of weight addition recorded in *First jumps* (Fig. 2; Table 1).

**Table 2:** The effects of *Jumping experience* (*JE*) and *Additional weight (AW)* treatments on the adjustments of (a) *Angular leg speed*, (b) *Take-off angle*, and (c) *Take-off velocity* of the water striders between *First* and *Second jumps*.

Variables		Females		Males	
Dependent	Independent	Estimate ± SD	95% CI	Estimate ± SD	95% CI
(a) Angular leg speed difference	Intercept	4.92 ± 3.77	− 2.73, 12.06	− 2.45 ± 4.86	− 12.28, 6.82
	AW	− 4.82 ± 11.68	− 28.43, 17.75	0.99 ± 8.68	− 16.71, 17.44
	**JE**	− **17.60** ± **7.12**	− **32.13, **− **4.35**	− 4.75 ± 12.98	− 27.78, 23.08
	**AW × JE**	**29.94** ± **14.69**	**1.48, 59.26**	− 0.11 ± 16.31	− 33.87, 29.62
(b) Takeoff Angle difference	Intercept	7.48 ± 5.55	− 4.07, 17.65	− 8.98 ± 3.79	− 16.25, − 1.30
	AW	− 8.90 ± 19.61	− 50.67, 26.13	1.52 ± 9.61	− 18.41, 20.06
	JE	− 12.62 ± 10.95	− 33.98, 9.16	14.54 ± 14.77	− 12.78, 45.44
	AW** × **JE	40.05 ± 25.50	− 8.63, 91.91	− 11.84 ± 19.65	− 51.31, 26.16
(c) Takeoff Velocity difference	Intercept	0.07 ± 0.07	− 0.06, 0.21	− 0.05 ± 0.06	− 0.18, 0.07
	AW	− 0.18 ± 0.18	− 0.56, 0.16	− 0.03 ± 0.003	− 0.29, 0.22
	**JE**	− **0.26** ± **0.11**	− **0.48, **− **0.05**	0.02 ± 0.20	− 0.32, 0.44
	**AW × JE**	**0.54** ± **0.25**	**0.07, 1.04**	− 0.08 ± 0.004	− 0.60, 0.40

Adjustment is measured by a difference between the *Second* and *First jump* for each individual (Fig. 1). Bold texts indicate statistically significant effects. Results obtained from linear regression analysis using 10,000 bootstrap iterations. Estimated coefficient values and their standard deviations (SD) as well as 95% confidence intervals (95% CI) are shown for females and males separately. Additional statistical analyses are shown in Supplementary Materials PART 2. The table corresponds to Fig. 3.

**Figure 4:**
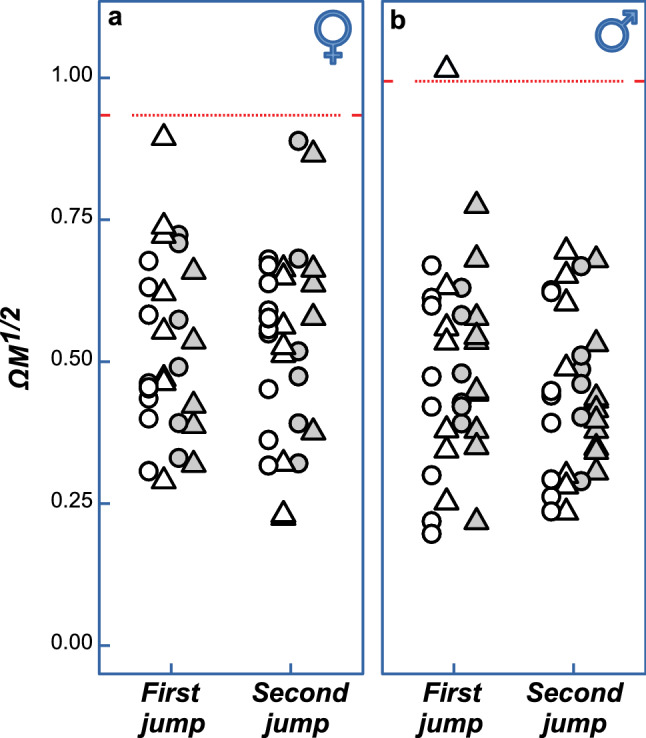
Jumps were performed within the physical constraint of the water surface tension properties. Comparison between the empirically observed jumping performance expressed in terms of the model-derived dimensionless index of angular leg speed ΩM^1/2^ and the physical constraint on performance expressed as the theoretical critical threshold value of ΩM^1/2^ beyond which surface breaking is expected (red horizontal lines near ΩM^1/2^ = 1). Triangles and circles indicate jumps in *jumping experience-present* and *jumping experience-absent* conditions respectively. Filled and open symbols indicate *weight-added* and *weight-not-added* conditions.

*Jumping behaviour and the physical constraints on performance*—In the theory of water strider’s surface-tension dominated jumps^3^, one dimensionless variable plays a crucial role: **ΩM**^1/2^. It is a complex variable and its full definition and formula are given in the Methods section: “Physical constraint from water surface: theoretical upper threshold of performance” . Its value increases as the *Angular leg speed*; *ω*), insect *body mass* (*m*), and the length of midleg sections that interact with water surface (combined length of midleg tibia and tarsus referred to here as the *wetted leg length*; *l*_*s*_), and increase. A water strider of a given body mass and leg morphology (i.e. when body mass and wetted leg length are constant) can affect the value of the variable **ΩM**^1/2^ by controlling the *Angular leg speed* during jump. The theory^3^ predicts that for a given total length of midlegs (*L*; see details in the Methods section) there is a threshold value of **ΩM**^1/2^ above which surface breaking will occur and the jump will be inefficient. We calculated this critical threshold for males and females separately (see Methods for details) and compared it with the values of **ΩM**^1/2^ observed in our experiments. Although *Angular leg speed* varied among the treatments and among individuals, the water striders (except one individual) did not cross the theoretical threshold value of **ΩM**^1/2^ above which meniscus breaking is theoretically expected (Fig. 4). This suggests that the observed adjustments of *Angular leg speed* were kept within the theoretical limit set by the physical properties of water surface. It is notable that the males’ *Angular leg speeds* were much below the threshold value, and female’s *Angular leg speeds* were closer to the critical value.

Even though the behaviours of water striders were within the theoretical threshold values, meniscus breaking was occasionally observed (in 25% of jumps: 21 of 62 in females, 10 of 62 in males). We found no statistical evidence for the effect of *Additional weight* or *Jumping experience* on the occurrence of meniscus breaking either in *First* or *Second jump* (Table 3). The breaking, if happened, typically occurred in the very late stage of jumping when an individual had already reached, or was about to reach, the final take-off velocity, and the legs’ downward push against the water surface was completed. Therefore, it is not surprising that *Take-off velocity* was not statistically significantly affected by the presence of meniscus breaking either in *First* (Wilcoxon rank sum test: female, *W* = 120, *P* = 0.379; male, *W* = 10, *P* = 0.155) or in *Second jump* (Wilcoxon rank sum test: female, *W* = 122, *P* = 0.765; male, *W* = 89, *P* = 0.912).

**Table 3:** The effects of *Additional weight (AW)* and *Jumping experience (JE)* on the occurrence of meniscus breaking (see methods for description of the variable “*Meniscus breaking*”).

Sex	Meniscus breaking	*First jump*			*Second jump*				
		Additional weight (AW)			Additional weight (AW)				*P*
		Not added	Added	*P*	Not-added		Added		
					JE-absent	JE-present	JE-absent	JE-present	
F	Absent	14	8	1	7	5	4	3	1
	Present	6	3		5	3	2	2	
M	Absent	13	6	0.226	6	5	4	8	0.946
	Present	2	0		2	2	2	2	

The table contains results (*P* values) of Fisher’s exact test of 2 × 2 table in *First jump* and 2 × 4 table in *Second jump* conducted for each sex separately.

## Discussion

It is not surprising that *weight-added* individuals in their *First jump* had lower leg speed and lower upward body velocity*.* It is consistent with the idea that individuals with added weight, without sufficient experience yet, might have initially applied as strong muscular force as *weight-not-added* individuals did, regardless of whether they might or might not have been able to enhance jumping performance by doing so. The subsequent repeated experience of frequent jumps provided the individuals with an opportunity to experience the weight their legs support and to adjust their behaviour based on their jumping experience. The observed behavioural changes between *First* and *Second jumps* suggest that only the females changed their behaviour in body-weight-specific manner: increasing the leg speed and jump velocity when weight is added and decreasing them when weight is not added. This observation is consistent with the idea that the frequent jumping gave females an opportunity to repeatedly experience their body weight and their jump performance, and that they adjusted their jumping behaviour based on the experience. Such a female-specific ability is consistent with the expectations based on the sexually-selected difference between sexes in the frequency in which they experience changes in the weight their legs support while skating and jumping on the water surface. Females, unlike males, often experience a sudden increase of additional body mass when the male initiates mating (copulation and intermittent guarding may last for hours, or even days^22,23,31,32^, and greatly increases females’ predation risk^24–26^, or a sudden decrease in body weight when the male dismounts (or is dislodged). This situation may lead to the evolution of females’ adaptations for adjusting their leg movements depending on whether they perceive light or heavy body weight. Body weight should have been better perceived when water striders had sufficient jumping experience (i.e. in *Jumping experience present* treatment) than during typical slow locomotion in a quiet experimental pool (i.e. in *Jumping experience absent* treatment), where they often remained motionless for extended periods of time especially during and soon after a feeding bout. The results are consistent with the hypothesis that without jumping experience the perception of body weight was imprecise and therefore we did not observe body-weight specific behavioural changes of jumping. However, with frequent jumping the perception of body weight by the individuals was more precise and therefore we observed weight-specific adjustments: the females that acquired (through frequent jumping) the perception of being relatively light changed their jumping behavior differently than the females who acquired through jumping the perception of being relatively heavy.

Females without additional weight significantly decreased *Angular leg speed* and *Take-off velocity* but only if they experienced jumping. Considering that all males also showed similar, albeit non-significant, tendencies towards decreasing the angular downward leg speed and the upward body velocity after experiencing frequent jumping, some common processes might have acted towards decreasing jumping performance over time in our test animals who experienced frequent jumps. However, this was not the case for the *weight-added* females. The significant interaction effects (*Additional weight* x *Jumping Experience)* indicated that over the three days of experiencing frequent jumps the *weight-added* females might have overcame these effects through weight-specific adjustments of locomotory behaviour in the opposite direction to this trend of decreasing jumping performance. These results are consistent with the hypothesis, that the *weight-added* females might have been able to detect the increase in their weight and the associated decrease in their locomotory performance only after they experienced frequent jumping. The results showed that those adjustments led to a restored performance. Currently, it is not entirely clear what might have been those common processes towards decreasing jumping performance over time in our test animals who experienced frequent jumps. Especially, why the *weight-not-added* females who experienced frequent jumping significantly decreased their leg speed after experiencing frequent jumping. Fatigue is one possibility. Although female water striders could bear the weight that is over 200% of their own mass^24^, which is heavier than the weights used in the experiment, fatigue could have built up over time and could have contributed to the observed decrease in *Angular leg speed* and *Take-off velocity*. If this was the main factor we should have observed the slowing down effect to be stronger in weight-added females because fatigue is expected to build up faster for jumping with an additional weight. However, this was not the case, indicating that fatigue cannot explain the observed pattern. Additionally, a repeated threat may induce habituation to the stimulus in animals leading to a decreased antipredatory response^33,34^. Hence, it is also possible that habituation to the repeated jump-invoking procedure, that might have been perceived as threatening, contributed to the decrease in take-off velocity over time in *weight-not-added* females as well as to some extent (non-significant) in all males. This effect should have led to a similar decrease in *Take-off velocity* over time in *weight-not-added* and *weight-added* females. This was not the case, and this explanation cannot account for the observed results. However, we cannot exclude that these two hypothetical effects occurred in both, *weight-not added* and *weight-added* females, and that the *weight-added* females overcame these effects through weight-specific adjustments of locomotory behaviour over the three days of experiencing frequent jumps.

Males did not show significant weight-specific changes in their jumping behaviour, which may suggest that predation-mediated natural selection for the ability to adjust the jump to the body weight may be weaker in males. This is expected because males do not experience frequent changes in their weight and because they are exposed to lower risk of predation^24,25^. Additionally, males’ jumping behaviour was far below the theoretical threshold where the meniscus breaking occurs and the jump becomes less efficient in escaping predation. Therefore, even if they lack the ability to modify the leg movements to their body mass, they may mostly stay below that critical threshold. We hypothesize that the relatively larger distance to the critical threshold visible in Fig. 4 is related to their smaller size and consistent with published data: Yang et al.^3^ have realized that the smaller water strider species (*G. comatus*, *G. latiabdominis*; Fig. 4 in^3^) are more distant in their jump performance from the threshold, and speculated why this may be the case. Here we see this trend between lighter males and heavier females within one species (*G. latiabdominis*).

The theoretical threshold of the downward leg speed is an outcome of the physical properties of water, which imposes constraints on the water striders’ jumping performance^2,3^. In the optimal jump, the leg downward movement in the theoretical model should not exceed the critical threshold angular velocity associated in the model with meniscus breaking, which dramatically decreases jumping performance^3^. Although the angular speed of legs in nearly all the jumps (except one individual) was indeed below the critical threshold determined from our modifications of the theoretical model of Yang et al.^3^ (explained in Methods), the breaking of the meniscus occurred in some jumps (Table 3) at the late stage of jumping when the individual had already accumulated most of the momentum that shaped the take-off velocity. Therefore the observed meniscus breaking did not affect the jump performance, and did not invalidate the theoretical threshold model. We can provide two hypothetical reasons for the observed meniscus breaking. Usually, the tibia of the water striders was bent during the jump, which prevents the meniscus breaking^35^. However, in some jumps it seems that the tibia did not curve enough, which resulted in meniscus breaking by the tip of the midleg just before the critical depth was reached. In other cases, the model assumptions of symmetrical simultaneous downward movements of left and right legs were not fulfilled, in which cases the average meniscus depth (average of the depths of the left and the right legs was used in calculations of the theoretical critical threshold of leg angular speed) did not correctly represent the depth of the leg that moved deeper and broke the water surface because it pushed the meniscus beyond the critical depth.

In summary, we provide empirical evidence that semi-aquatic insects are able to use personal experience during locomotion (jumping) to adjust their locomotory performance in response to the changes in body weight, and that they do it within the constraints set by the physical properties of environment (physics of water surface). Hence, in addition to reacting to changes of their physical circumstances such like changes in body weight, water striders are able to deal with the relatively dramatic physical constraint on jump biomechanics. While the behavioral ability to match locomotion to physical or biological circumstances has been known in other insects [e.g. 36–40], this is the first demonstration that this ability can be acquired through repeatedly experiencing the circumstances. This behavioral ability of short-term changes through experience is reminiscent of developmentally acquired (long-term) modifications of jump biomechanics in response growing up in environments of different predation risk^41^. The behavioural adjustments through personal experience were statistically significant in females. We propose that the observed difference between the sexes in the use of individual experience to adjust their locomotory performance to the changing body weight may be a female-specific adaptation to frequent mating that involves males riding on the back of females. As both sexes across different species seem to match their leg movements to their morphology during jumps^3^, our results suggest that the fine adjustments from experience observed in our experiments are not the sole mechanism that contributes to the optimal species-specific match between morphology and leg movements. This mechanism is likely to play a role in females of many water strider species because their mating behaviour is similar to our study species^21–32^. These findings allow us to think about a biologically inspired scheme to make robots learn to optimize their performance using repeated experience based on physical or computational intelligence^42^.

## Methods

### Study animals

Between June and August 2014, male and female *Gerris latiabdominis* were collected using insect nets from small ponds and an old swimming pool at Seoul National University, Seoul, South Korea. The number of water strider collected weekly varied depending on the current experimental requirements. In total, we collected near 100 individuals and used 62 of them in the experiments reported here. Collected water striders were housed in plastic containers (52 × 42 × 18 cm, 2–4 individuals/container) with aerated water, foam resting platforms, and two frozen large crickets per container per day. Each water strider’s thorax was marked with three unique color-coded dots using enamel paints. Females and males were housed separately.

### Experiments

#### Effect of weight addition on the performance of first jumps

Experimental design is graphically summarized in Fig. 1b (also see Supplementary Materials PART 4). To determine the effect of increase in body mass on the behaviour, we tested water striders in two conditions of the *Additional weight* treatment: *weight-added* (11 females and 16 males) and *weight-not-added* (20 females and 15 males). After measuring the weight with an Ohaus electronic scale with the precision of 0.1 mg, the water striders were randomly assigned to either of the treatment group. In *weight-added* group, a flat coiled aluminum wire (~ 7.5 mg weight in males, ~ 10.5 mg weight in females) was secured to the backs of water striders with a tiny drop of non-water soluble glue gel (applied only on top of thorax). Added weights caused an increase of body mass by about 50% (54.5% ± 9.2 (mean ± SD) in males and 52.8% ± 5.7 in females). The body weight of a male is about 70% of female body weight on average (similar based on median or average body weights). Preliminary theoretical calculations using the model of surface-tension dominated jumping^3^ suggested that an average female with the extra weight equivalent to the average male body mass would be able to jump and to achieve take-off velocity of about 0.75 m/s for the leg angular velocity of about 40 rad/s. However, based on our observations, when a male sits on the female’s back during copulation and mate guarding the male’s hindlegs are always on the water, probably adding to the support for the mating pair on the water surface. The tips of male midlegs can also be on the water surface, possibly also helping in support on the water surface. All evidence suggests that the male’s support on the water surface contributes to some extent to the forces maintaining the mating pair on the surface of water. Hence, the female does not perceive the full body weight of the mating male. Our preliminary trials with additional weight of different masses indicated that the weight similar to the male body mass is too heavy for the purpose of our experiments because some females were not able to stay on the surface for extended time periods. This was not observed for the extra weights used in our experiments.

After the weight was added to the water strider, the animal was allowed to rest with 2–3 other individuals of the same sex in a container filled with water (20 × 14 × 10 cm). After three hours, the water strider was placed in a box where the 3-D slow motion movie of the jump was recorded (labeled as the *First jump*; see below for the details). In *weight-not-added* group the individuals were treated similarly and handled for similar duration but no extra weight (neither wire nor the glue) was put on their backs. Triggering repeated jumps successively many times in the small container in which they were filmed likely leads to changes in performance due to repeated jumps within relatively short time and due to accumulated effect of the heat of the lights needed for high speed filming. We decided to use the design in which we took one jump per individual. The final sample sizes differ between treatments because some movies were discarded at the analysis stage for technical reasons.

#### Effect of jumping experience on adjustment of jumping performance

In order to test the effect of individual’s experience on adjustments of jumping behaviour we subjected the males and females from the preceding *First jump* to two conditions of *Jumping experience [JE]* treatment: *presence* and *absence* of frequent jumping during a three-day period (Fig. 1). For three days following the filming of *First jump*, water striders were kept in groups of 3–4 individuals per container (20 × 14 × 10 cm; filled with aerated water) and fed two frozen crickets per day. Each container was assigned to either *JE-present* or *JE-absent* treatment. In the former, we used an aluminum wire bent in the shape of a hook to touch or poke the insect’s underside in order to trigger 3–5 jumps/hour over 5 h/day. Jumps provide individuals with repeated experience of their jumping performance and the opportunity to adjust jumping behaviour. In the latter, individuals were not exposed to these procedures. At the end of the three days, the jumps (*Second jump*) were recorded in the same manner as for *First jump*. Sample sizes (listed in caption to Fig. 3 and in Supplementary Table 7 in Supplementary Materials PART 4) differ between treatments because some movies were discarded for technical reasons (see below) and some animals escaped or died.

### High speed filming of jumps

We used three synchronized high-speed cameras (FasTec Troubleshooter Model #: TS1000ME), with lens axes perpendicular to one another (Supplementary Fig. 2b in Supplementary Materials PART 4). Lights (Photon Super Energy Light, Aurora CCD-250 W, and PLTHINK Photo Light Think with Metal Halide bulbs) were placed directly opposite to each camera lens (Supplementary Fig. 2b). At the center of the setup was a 10 × 10 × 10 cm clear Plexiglas box filled with water. The jumps were invoked by an aluminum wire bent in the shape of a hook underneath the water surface. Jumps were recorded at 500 frames per second. Clips with insects that were accidentally pushed upward by the wire were excluded from the analyses. Examples of jumps extracted for the movies are shown in the Supplementary Movie.

### Variables extracted from the videos

We tracked the locations of body parts of water striders frame by frame in a three dimensional *x, y, z*, coordinate system (*x, y* are horizontal axes, *z*-coordinates are on the vertical axis, and origin is located at the level of undisturbed water surface) using video tracking software MaxTRAQ 3D (Innovision Systems). We tracked three markers; body center (defined as point between midleg and hindlegs), right midleg dimple depth, and left midleg dimple depth. Dimple depth is the deepest point of water surface deflection under the pressure from a midleg. From this data we calculated upward (vertical) and forward (horizontal) body velocities. For each pair of consecutive frames, we calculated the raw upward velocity of body center (along the vertical axis *z*) by dividing the vertical shift of body center (vertical distance between *z* coordinates of body center in the two consecutive frames) by the duration (2 ms between frames in 500 fps movie). Then, we calculated *smoothed vertical velocity* (m/s) by using rolling three-point average of three successive velocities. In an analogical manner we calculated the values of *smoothed horizontal velocity* (m/s) during a jump. From the data we extracted four variables used in analyses:

*Angular leg speed* (rad/s): Legs move downward as a result of downward angular femur movement powered by insect’s muscles, and the rotational rate of the leg downward movement is termed *Angular leg speed* (rad/s). To match the angular leg speed calculations in the theoretical model^3^, we calculated the *Angular leg speed* in several steps using empirical data and theoretical formulas from the existing model^3^. The coordinate system included vertical axis (*z*) with origin (*z* = 0) at the level of undisturbed water surface. First, for each frame we calculated *average dimple depth* as an average *z* from the left and right dimple depths’ *z* values, and the *downward leg reach* as the distance between body center’s *z* and the *average dimple depth*. Then, for each pair of consecutive frames, we calculated the *downward velocity* of dimple depth relative to body center (along the vertical axis *z*) by dividing the change in the *downward leg reach* between two consecutive frames by the duration (2 ms between frames in 500 fps movie). By using rolling three-point average from three successive *downward velocities* we obtained *smoothed leg speed* (m/s). Finally, we calculated the *maximal downward speed of legs v*_*s,max*_ (m/s) as an average from the three largest *smoothed velocity* values. The downward *Angular leg speed* (*ω*) was calculated according to Yang et al.^3^ by approximation starting from the previously approved formula^3^ for the *maximal downward speed of legs v*_*s,max*_ containing leg length *l*_*l*_: $v_{s,\max } \approx \omega *\left( { l_{l} - y_{i} } \right)*\sin \left( {2\omega t} \right)$ (*y*_*i*_ indicates distance from the surface to insect body at rest and *t* indicates time during jump). See “Physical constraint from water surface: theoretical upper threshold of performance” below for more details about the model. The calculations resulted in the variable (*Angular leg speed*) that was directly relevant to the theoretical predictions of the optimal jumping behaviour^3^.

*Take-off angle* (deg): We defined *take-off angle* (deg) as the angle of trajectory to the water surface when the water strider leaves the surface of water. Takeoff angle was calculated from the ratio of horizontal and vertical vectors of the smoothed body center velocities.

*Take-off velocity* (m/s): *Take-off velocity* (m/s) is the vertical velocity of body center when the water strider leaves the surface of water. We determined the moment of leaving the water surface as the frame when legs disengage from the surface. Vertical velocity indicates how fast the animal removes itself from surface of water. A high take-off velocity is important when predators attack from underneath the water surface. This variable is a crucial component of the theoretical model of optimal jumping performance by water striders^3^.

*Meniscus breaking* (binary): Sometimes jumping water striders break the water surface. When left or right midleg pierced the water surface by more than a quarter of its full leg length the jump was categorized as a jump with *meniscus breaking-present*. Otherwise the jump was categorized as a jump with *meniscus breaking-absent*.

### Statistical analyses

*Effect of weight addition on jumping performance*—To analyze the effect of *Additional weight* on jumping performance of *First jumps*, we used Wilcoxon rank sum tests (Mann–Whitney test) to compare *weight-added* with *weight-not-added* groups for each sex separately. We used nonparametric statistical methods here because of small sample size that does not allow to confirm the parametric methods’ assumptions with high reliability (nevertheless the tests indicated that the parametric assumptions were probably met and in Supplementary Materials PART 1we also provide results from parametric comparisons: *t*-tests and Welch’s t-tests. In order to investigate whether *Additional weight* effect is statistically significantly different between sexes we switched to parametric analyses and run two-way ANOVA tests including the interaction effect between two independent variables (*Additional weight* and *Sex*) separately for the three dependent variables: *Angular leg speed*, *Take-off angle* and *Take-off velocity*.

*Effect of jumping experience on adjustments of jumping performance*—For each individual, we calculated three indices of adjustment (change) in performance between *First* and *Second* jumps. For each of the three dependent variables, we subtracted the value at *First jump* from the value at *Second jump* (for analysis of *Second jump* solely—see Supplementary Materials PART 3). Linear regression model was used to investigate the effect of *Jumping experience* and *Additional weight* treatments on jumping adjustments. Because of the small sample sizes, estimates and 95% confidence intervals of regression coefficients were reported using nonparametric bootstrap procedure with 10,000 replications of each linear model (‘boot’ package in R).

*Meniscus breaking—*To analyze the effect of *Jumping experience* and *Additional weight* treatments on the probability of breaking of the water surface (*Meniscus breaking-present*) we used Fisher’s exact test. To determine the effect of *Meniscus breaking* on *Take-off velocity* we used Wilcoxon rank sum test separately for males and females. All statistical analyses were performed using R (version 3.3.2;^43^).

### Physical constraint from water surface: theoretical upper threshold of performance

During a water strider’s jump, the water surface can be pushed downward only so much before breaking. Thus, a theoretical upper threshold of performance exists. The mathematical model of surface tension dominated jumping^3^ allows to predict the moment of surface breaking and the optimal behaviour of vertically jumping water striders without surface breaking. The model contains a non-dimensional variable: **ΩM**^1/2^. Its value depends, among others, on the *Angular leg speed* used by water striders during jump and on morphology: body mass and midleg’s tibia and tarsus length—the leg parts on which water strider’s body is supported on the water surface. The theory predicts that for a given total length of midlegs there is a threshold value of **ΩM**^1/2^ above which surface breaking will occur and the jump will be inefficient. We determined if water striders used *Angular leg speed* values that resulted in theoretical values of **ΩM**^1/2^ below this critical threshold. In order to more precisely predict the theoretical threshold value of **ΩM**^1/2^we modified the original model^3^. The original model used a simple average length of all four legs (mid-legs and hind-legs) and did not reflect a difference between the length of hind and mid legs. We changed the original equation into systems of differential equations using information about midlegs and hindlegs separately. Modified predicted threshold values of **ΩM**^1/2^ were compared with empirically observed values of **ΩM**^1/2^in order to determine if the observed adjustments of leg speed by water striders lie within the theoretical limit of performance set by physical properties of water. We used the same parameters as in^3^ for, *l*_*c*_, capillary length, *g*, gravitational acceleration, *ρ*, density of water. Because of the short length of legs, we approximated *C*, flexibility factor, as 1. Downward angular velocity of leg rotation, *ω*, was calculated by approximation, $v_{s,max} \approx \omega {\Delta }l\sin \left( {2\omega t} \right)$. The averaged length of femur, tibia and tarsus were measured from 24 individuals of each sex in *G. latiabdominis* and *y*_*i*_, the distance from body center to the undisturbed water surface in the resting position of the water strider was measured from 4 movie clips of each sex. Measured variables were averaged (Supplementary Table 8 in Supplementary Materials Part 4) and used to determine *l*_*t*_, average length of tibia plus tarsus, *l*_*l*_, average leg length, and Δ*l* = *l*_*l*_ − *y*_*i*_, maximal reach of the leg. Note that, the average length of tibia plus tarsus of hind and mid legs, *l*_*th*_, *l*_*tm*_, and the average length of maximal reach of leg of hind and mid legs, Δ*l*_*h*_, Δ*l*_*m*_, can be represented as:

$$l_{th} = 0.77l_{t} ,l_{tm} = 1.23l_{t} ,\Delta l_{h} = 0.84\Delta l,\Delta l_{m} = 1.16\Delta l$$

1 $$ \frac{{d^{2} H_{m} }}{{d\left( {\omega t} \right)^{2} }} + \frac{4 \cdot 1.23}{{{\Omega }^{2} M}}H_{m} \left( {1 - H_{m}^{2} /4} \right)^{1/2} + \frac{4 \cdot 0.77}{{{\Omega }^{2} M}}H_{h} \left( {1 - H_{h}^{2} /4} \right)^{1/2} - 2 \cdot 1.16L\cos \left( {2\omega t} \right) = 0, $$

2 $$ \frac{{d^{2} H_{h} }}{{d\left( {\omega t} \right)^{2} }} + \frac{4 \cdot 1.23}{{{\Omega }^{2} M}}H_{m} \left( {1 - H_{m}^{2} /4} \right)^{1/2} + \frac{4 \cdot 0.77}{{{\Omega }^{2} M}}H_{h} \left( {1 - H_{h}^{2} /4} \right)^{1/2} - 2 \cdot 0.84L\cos \left( {2\omega t} \right) = 0, $$

where $$H_{m} = h_{m} /l_{c} $$ is the dimensionless dimple depth of mid legs (*h*_*m*_, dimple depth of mid legs), $$H_{h} = h_{h} /l_{c}$$ is the dimensionless dimple depth of hind legs (h_1_, dimple depth of hind legs), $${\Omega } = \omega \left( {l_{c} /g} \right)^{1/2}$$ is the dimensionless angular velocity of leg rotation, $$M = m/\left( {\rho l_{c}^{2} Cl_{t} } \right)$$ is the dimensionless index of insect body mass (*m*, insect body mass), and $$L = {\Delta }l/l_{c}$$ is the dimensionless maximum downward reach of leg. The variable ***ΩM***^1/2^ is calculated, as the name suggests, by multiplying the above-defined Ω by square root of *M*^3^. For a given *L*, the optimal value of ***ΩM***^1/2^ for maximal take-off velocity is achieved when the maximal *h*_*m*_ is equal to the critical depth, $$\sqrt 2 l_{C}$$, just before meniscus breaking. Wetted leg length, *l*_*s*_, was measured from 24 individuals of each sex in *G. latiabdominis* and initial height, *y*_*i*_*,* was measured from 12 recorded videos of females (6 individuals) and 9 recorded videos of males (5 individuals) (Supplementary Table 8 in Supplementary Materials Part 4). The *ode45* function in Matlab was used to solve Eqs. (1) and (2) to get the optimal ***ΩM***^1/2^ of male and female water striders (red lines in Fig. 4).

## Supplementary information

Supplementary Information 1.

Supplementary Video 1.

Supplementary Video 2.

## Data Availability

Data needed to evaluate the conclusions in the paper are present in the paper and/or the Supplementary Materials. Additional data related to this paper may be requested from the authors.
