# Supplementary material for: Water strider females use individual experience to adjust jumping behaviour to their weight within physical constraints of water surface tension
Source: Sci Rep. 2020 Oct 29;10:18657. doi: 10.1038/s41598-020-75564-x (PMC7596521; doi:10.1038/s41598-020-75564-x)
Supplement: Supplementary file 1 — Supplementary Information 1. [file 41598_2020_75564_MOESM1_ESM.pdf]

SUPPORTING INFORMATION TO:

Water strider females use individual experience to adjust jumping behaviour to their weight within physical constraints of water surface tension

Short title: **Water striders adjust jumping through experience**

Minjung Baek [1,2], Katherine M. Lawin [3], Christina J. Codden [4], Hangkyo Lim [3,5], Eun-jin Yang [6,7], Ho-young Kim [6,7], Sang-im Lee [8,1 *], Piotr G. Jablonski [1,9 *]

1 Laboratory of Behavioral Ecology and Evolution, School of Biological Sciences, Seoul National University, Seoul 151-747, S. Korea;
2 University of Arizona, EEB, Tucson, USA
3 University of St. Thomas, 2115 Summit Ave., St. Paul, MN 55105 USA ;
4 Northeastern University, 360 Huntington Avenue, Boston, MA 02215, USA ; 5 Notre Dame of Maryland University, 4701 North Charles St, Baltimore, MD 21210 USA ;
6 Department of Mechanical and Aerospace Engineering, Seoul National University, Seoul, South Korea;
7 Institute of Advanced Machines and Design, Gwanak-ro 1, Gwanak-gu, Seoul 151-742
8 School of Undergraduate Studies, Daegu-Gyeongbuk Institute of Science and Technology, Daegu, South Korea
9 Museum and Institute of Zoology PAS, Wilcza 64, Warsaw, Poland

[*] corresponding authors:
Sang-im Lee: sangim@dgist.ac.kr;
Piotr G. Jablonski: snulbee@behecolpiotrsangim.org;
Ho-young Kim: : hyk@snu.ac.kr;

Supplementary Materials PART 1 1
Supplementary Materials PART 2 5
Supplementary Materials PART 3 7
Supplementary Materials PART 4 9
Supplementary Videos explanations...11

SUPPLEMENTARY MATERIALS: PART 1

Effect of additional weight on jumping performance in the *first jumps*

Wilcoxon tests

(this text is relevant to Fig.2 in the main text)

In order to test the effect of added weight on the behaviour at the *First jumps* that were recorded shortly after weight addition procedure, we measured water striders' ***Angular leg speed*** (rad/s; variable names are in bold italic; detailed explanations for the variables are in the Methods section) of downward mid-leg movement during jump, ***Take-off angle*** (deg; angle between body center trajectory and water surface at take-off) and the vertical ***Take-off velocity*** (m/s) for two conditions of the ***Additional weight*** treatment: *weight* (~50% of body mass)-*added* vs. *weight-not-added*. Although the ***Angular leg speed*** of the *weight-added* females was marginally non-significantly slower than that of the *weight-not-added* females (Fig. 2a in the main text; Wilcoxon test: $W = 150$, $P = 0.104$), the trajectories of the *weight-added* females were significantly less steep (Fig. 2b in the main text; Wilcoxon test: $W = 162$, $P = 0.038$), which was associated with significantly lower ***Take-off velocity*** (Fig. 2c in the main text; Wilcoxon test: $W= 172$, $P < 0.01$) of the *weight-added* females. Respective comparisons for males (Fig. 2d, e, f in the main text) revealed no significant differences in response to weight addition (Wilcoxon tests: ***Angular leg speed***: $W = 143$, $P = 0.379$; ***Take-off angle***: $W = 109$, $P = 0.682$; ***Take off velocity***: $W = 152$, $P = 0.216$), but showed similar trends in ***Angular leg speed*** and ***Take-off velocity*** (Fig. 2d-f in the main text). Comparisons of visually perceived (regardless of statistical significance) differences in Fig. 2a with Fig. 2d and Fig. 2c with Fig. 2f indicate that *weight-added* individuals of both sexes used lower ***Angular leg speeds*** and jumped at lower ***Take-off velocities*** than *weight-not-added* ones. This difference appeared to be stronger in females especially for ***Take-off velocity*** (Fig. 2c and f).

t-tests

(this text is relevant to Fig.2 in the main text)

In addition to the Wilcoxon rank sum (Mann-Whitney) test, we also used parametric statistics for the comparisons between *weight-added* and *weight-not-added* treatments for males and females separately. When the variances were not equal between weight added treatments then the appropriate version of the t-test (Welch's for samples of different variance) was used for these parametric comparisons between *weight-added* and *weight-not-added* groups in 1st jump (Supplementary Table 1). The *P* values from *t*-tests are slightly higher than for the equivalent *P* values form the Wilcoxon rank sum (Mann-Whitney) test possibly due to higher sensitivity of *t*-test to outliers. This resulted in one slight discrepancy in formal statistical conclusions for the ***Take-off angle*** in females: while the Mann-Whitney test showed a significant ($P<0.05$) difference between additional weight treatments (Fig. 2 in the main text; "Wilcoxon tests" above) the *t*-test suggested marginally non-significant effect ($P<0.062$). Additionally, the column "Tukey test" in the Supplementary Table 1 contains *P* values from the complete set of Tukey multiple comparisons of all means based on 95% family-wise confidence interval for ANOVA conducted for each sex separately (Supplementary Table 2).

Supplementary Table 1. *t*-test results for comparisons between *weight-added* and *weight-not-added* individuals for males and females separately in their *First jump* presented in Supplementary Fig.1. Additionally, Tukey *P* values extracted from Supplementary Table 3 are shown here for convenience.

Variable	Females				Males			
	Student t-test			*Tukey test*	*Student t-test*			*Tukey test*
	t	df	*P* value	*P* value	t	df	*P* value	*P* value
(a) Angular leg speed	1.8196	29	0.0792	0.4109	1.259	22.114*	0.2212	0.4792
(b) Takeoff angle	1.9424	29	0.0619	0.1795	-0.6213	29	0.5392	0.9370
(c) Takeoff velocity	2.7748	29	0.0096	0.0407	1.1618	29	0.2548	0.6414

Tukey tests

(this text is relevant to Fig. 2 in the main text)

To investigate whether ***Additional weight*** effect is statistically significantly different between sexes we switched to parametric analyses after confirming that the assumptions of ANOVA were formally met (however due to small sample size this conclusion must be treated with caution). For ***Angular leg speeds*** and ***Take-off velocities***, no such interactions were significant indicating that the difference between *weight-added* and *weight-not-added* conditions appeared similar for males and females (Table 1 in the main text). However, for ***Take-off angle***, the interaction term ***Sex * Additional weight*** was only marginally non-significant at $P = 0.064$ indicating a possibility of a population-wide different effect of added weight on the trajectory's steepness for males than females. In supplementary Table 2 below, we report here full sets of Tukey comparisons that followed after the ANOVA analysis presented in Table 1 of the main text.

Supplementary Table 2. The full set of Tukey comparisons for *First jump* conducted in association with the ANOVA analyses presented in Table 1 of the main text and related to Fig.2. F:Y indicates females with additional weight added (Additional weight: Yes); F:N indicates females without additional weight (Additional weight: No); M:Y indicates males with additional weight added (Additional weight: Yes); M:N indicates males without additional weight (Additional weight: No).

Variable	Comparison	Difference	95 % confidence limits lower	upper	Adjusted *P* value
Angular speed					
	F:Y-F:N	-9.5638	-25.8104	6.6828	0.4109
	M:Y-M:N	-8.4764	-24.0313	7.0786	0.4792
	M:Y-F:Y	4.1683	-12.7837	21.1202	0.9150
	M:N-F:N	3.0808	-11.7023	17.8640	0.9458
	M:Y-F:N	-5.3955	-19.9123	9.1212	0.7596
	F:Y-M:N	-12.6446	-29.8252	4.5360	0.2203
Take-off angle					
	F:Y-F:N	-14.0971	-32.2239	4.0296	0.1795
	M:Y-M:N	3.8209	-13.5342	21.1759	0.9370
	M:Y-F:Y	9.9868	-8.9269	28.9004	0.5065
	M:N-F:N	-7.9313	-24.4252	8.5627	0.5843
	M:Y-F:N	-4.1104	-20.3071	12.0863	0.9075
	F:Y-M:N	-6.1659	-25.3347	13.0030	0.8299
Take-off velocity					
	F:Y-F:N	-0.2520	-0.4962	-0.0078	**0.0407**
	M:Y-M:N	-0.1044	-0.3382	0.1295	0.6414
	M:Y-F:Y	0.0994	-0.1554	0.3542	0.7317
	M:N-F:N	-0.0482	-0.2705	0.1740	0.9394
	M:Y-F:N	-0.1526	-0.3708	0.0656	0.2611
	F:Y-M:N	-0.2037	-0.4620	0.0545	0.1696

The most robust conclusion from all these comparisons is that *weight-added* individuals have slower vertical ***Take-off velocity*** than the *weight-not-added* individuals. The second reasonable conclusion is that *weight-added* females, but not males, tend to have less steep take-off trajectories than the *weight-not-added* females.

Additionally, there are indications (Wilcoxon tests and *t* tests), albeit not formally confirmed by significant “Sex : Weight Added” interaction terms in ANOVA (Table 1 of the main text), that females were more affected by the additional weight. As a potential mechanism for the reduced jumping performance of the weight-added females in their first jumps, we hypothesize that the prominent slow-down of vertical jumping may be a female-specific strategy that evolved as an adaptation to frequent experiences of an abrupt change in body weight due to a presence of a male on a female’s back during mating. Lower ***Take-off velocity*** in *weight-added* females was also associated with less steep jump trajectory (lower ***Take-off angle***). Thus, by being “careful”, i.e. moving the legs slowly, jumping less vertically and slowly, the females, who face an increased risk of predation immediately

after males mount them, may decrease the probability of surface breaking (which increases the chance of jumping failure and thus greatly increase the risk of being captured by a predator) before they learn how to adjust their jumps through personal experience. The lack of difference in surface breaking frequency (Table 3 of the main text) between *weight-added* and *weight-not-added* females in *First jump* may indicate that this strategy was successful in preventing the increase of surface breaking when the body became heavier due to male’s mounting.

SUPPLEMENTARY MATERIALS: PART 2

Supplementary Table 3. Results of one sample Wilcoxon rank median test (two-tailed) of the null hypothesis that median value of a variable equals zero for the three variables indicating behavioural change during the three days between *First* and *Second jumps*. The *P*-values are not corrected for multiple comparisons. They are used here only as additional indicators of the detailed differences that are behind the significant interaction (between *Jumping Experience* and *Additional Weight* in females; see main text, Table 2 and Fig.3), which provides the sole basis for our main conclusions. Additionally, we provide the Bonferroni-sequentially corrected [1, 2, 3] values in italic in parentheses.

Variables	Group category		Females		Males	
	Weight	Jumping experience	V statistic	p-value	V statistic	p-value
(a) Angular leg speed difference (rad/s)	Not-added	Absent	58	0.151 *(0.389)*	14	0.641 *(0.954)*
		Present	4	0.055 *(0.202)*	13	0.938 *(0.996)*
	Added	Absent	10	1.000 *(1.000)*	11	1.000 *(1.000)*
		Present	12	0.313 *(0.527)*	13	0.160 *(0.503)*
(b) Take-off angle difference (deg)	Not-added	Absent	56	0.204 *(0.495)*	6	0.109 *(0.371)*
		Present	15	0.742 *(0.934)*	14	1.000 *(1.000)*
	Added	Absent	10	1.000 *(1.000)*	7	0.563 *(0.809)*
		Present	14	0.125 *(0.414)*	15	0.232 *(0.548)*
(c) Take-off velocity difference (m/s)	Not-added	Absent	48	0.519 *(0.768)*	15	0.742 *(0.742)*
		Present	4	0.055 *(0.202)*	11	0.688 *(0.902)*
	Added	Absent	8	0.688 *(0.688)*	7	0.563 *(0.916)*
		Present	12	0.313 *(0.675)*	15	0.232 *(0.653)*

Supplementary Table 4. Mann-Whitney test comparisons of behavioural changes between *JE-absent* and *JE-present* ("***Absent* ⇔ *Present***") treatment for *weight-not-added* and *weight-added* separately. This table is relevant to the results shown in Fig. 3 of the main text. The *P*-values are not corrected for multiple comparisons: they are used here only as additional indicators of the detailed differences that are behind the significant interaction (between ***Jumping Experience*** and ***Additional Weight*** in females; see main text: Table 2 and Fig. 3), which provides the sole basis for our main conclusions.

Variables	Pairwise comparisons ("⇔" indicates compared treatments)		Females		Males	
	Additional Weight	Jumping experience	W	*P*-value	W	*P*-value
(a) Angular leg speed difference	*Not-added*	*Absent ⇔ Present*	79	0.016	36	0.397
	Added	*Absent ⇔ Present*	12	0.662	39	0.368
(b) Takeoff angle difference	*Not-added*	*Absent ⇔ Present*	63	0.270	24	0.694
	Added	*Absent ⇔ Present*	8	0.247	32	0.875
(c) Takeoff velocity difference	*Not-added*	*Absent ⇔ Present*	74	0.047	35	0.463
	Added	*Absent ⇔ Present*	10	0.429	37	0.492

SUPPLEMENTARY MATERIALS: PART 3

Jumping performance in *Second jump* is the outcome of the initial jumping performance and the behavioural adjustments that individuals made between *First* and *Second* jumping trials. There was no noticeable overall significant effect of ***Jumping Experience*** and ***Additional weight***, or interaction between them, on jumping performance for females and males. (Supplementary Table 5). Nevertheless, Mann-Whitney tests (Supplementary Table 6) showed that the only significant difference *between weight-added* and *weight-not-added* individuals was observed for females' ***Take-off velocity*** in ***Jumping Experience*** *absent* treatment: the difference was similar to the initial difference in the *First jump* (compare Figure 2c in the main text with ***Jumping*** *Absent* in Supplementary Figure 1c), confirming the main conclusion that only the females who experienced frequent jumping adjusted their behaviour in body-weight specific manner such that after adjustment the initial difference between weight-added and weight-not-added females disappeared. For male water striders, significant differences in ***Take-off velocity***, ***Take-off angle*** and ***Angular leg speed*** were not found regardless of the jumping experience or additional weight treatments (Supplementary Table 5, Supplementary Table 6).

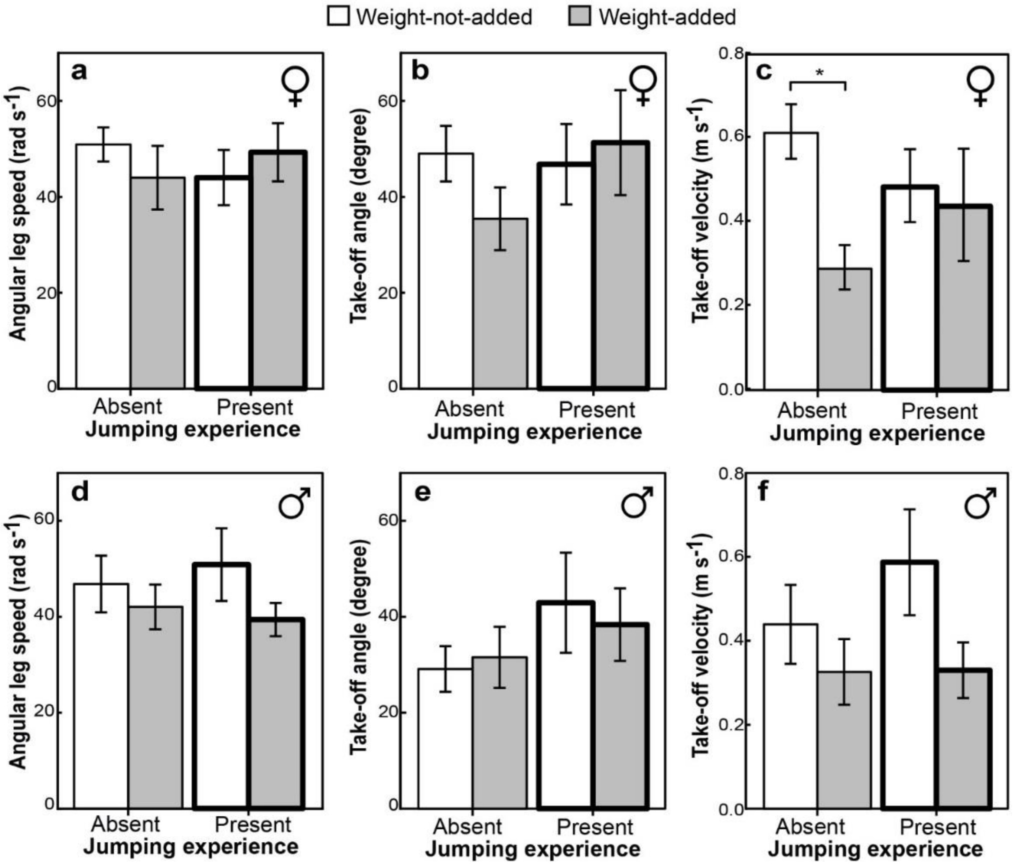

Supplementary Figure 1. The effects of ***Additional weight*** (gray-filled boxes indicate *weight-added* and unfilled boxes indicate *weight-not-added* treatments) and ***Jumping experience*** (thin border boxes indicate *absence* and thick border boxes indicate *presence* of additional jumping experience) on jumping performance of *G. latiabdominis* females (a-c) and males (d-f) in *Second jumps*. Average (error bars indicate Standard Error) ***Angular leg speed*** (a, d), ***Take-off angle*** (b, e), and ***Take-off velocity*** (c, f) for each treatment group are shown. Statistical results are in Supplementary Table 4. Sample sizes in a-c in order from left to right in each panel: 12, 8, 6, 5. Sample sizes in d-f in order from left to right in each panel: 8, 7, 6, 10.

Supplementary Table 5. The effect of ***Jumping experience*** (JE) and ***Additional weight*** (AW) treatments on the ***Angular leg speed***, ***Take-off angle***, and ***Take-off velocity*** of males and females in *Second jumps*. The table corresponds to Supplementary Fig.1. Results are obtained from linear regression analysis using 10,000 bootstrap iterations. Estimated coefficient values and their standard deviations (SD) as well as 95% confidence intervals (95% CI) are shown for females and males separately.

Variable		Females		Males	
Dependent	Independent	Estimate (SD)	95% CI	Estimate (SD)	95% CI
(a) Angular leg speed	Intercept	50.91 (3.52)	43.92 ~ 57.65	46.80 (5.79)	35.83 ~ 58.62
	AW	-6.90 (7.50)	-20.77 ~ 8.99	-4.77 (7.35)	-19.472 ~ 9.242
	JE	-6.90 (6.76)	-20.26 ~ 6.34	4.04 (9.48)	-14.658 ~ 22.483
	AW * JE	12.20 (11.10)	-10.25 ~ 33.71	-6.69 (11.05)	-28.148 ~ 15.375
(b) Take-off angle	Intercept	49.01 (5.72)	38.01 ~ 60.40	29.11 (4.71)	19.67 ~ 38.33
	AW	-13.58 (8.69)	-30.43 ~ 3.37	2.43 (7.93)	-12.84 ~ 18.55
	JE	-2.22 (9.98)	-22.22 ~ 16.54	13.82 (11.34)	-9.08 ~ 36.23
	AW * JE	18.09 (16.10)	-14.72 ~ 48.39	-6.99 (14.98)	-35.77 ~ 23.07
(c) Take-off velocity	Intercept	0.61 (0.07)	0.48 ~ 0.74	0.44 (0.09)	0.25 ~ 0.62
	AW	-0.32 (0.09)	-0.48 ~ 0.15	-0.11 (0.12)	-0.35 ~ 0.13
	JE	-0.13 (0.11)	-0.34 ~ 0.08	0.15 (0.16)	-0.17 ~ 0.45
	AW * JE	0.28 (0.18)	-0.07 ~ 0.64	-0.14 (0.19)	-0.51 ~ 0.23

Supplementary Table 6. T Wilcoxon rank sum (Mann-Whitney) tests in the *Second jump. Italic* indicates the treatments that are compared in each row. Bold marks the significant result.

Variables	Pairwise comparisons		Females		Males	
	Jumping experience	Weight	W statistic	p-value	W statistic	p-value
(a) Angular leg speed	*Absent/Present*	Not-added	58	0.4727	24	0.6943
	Absent/Present	Added	12	0.6623	37	0.4923
	Absent	*Not-added/ Added*	46	0.3845	28	0.662
	Present	*Not-added/ Added*	19	0.9433	44	0.4173
(b) Takeoff angle	*Absent/Present*	Not-added	51	0.8506	21	0.4634
	Absent/Present	Added	8	0.2468	26	0.7128
	Absent	*Not-added/ Added*	51	0.1797	21	0.7546
	Present	*Not-added/ Added*	18	0.8329	37	0.8868
(c) Takeoff velocity	*Absent/Present*	Not-added	64	0.238	18	0.281
	Absent/Present	Added	11	0.5368	31	0.9578
	Absent	*Not-added/ Added*	**66**	**0.003232**	30	0.4908
	Present	*Not-added/ Added*	24	0.6216	53	0.08782

SUPPLEMENTARY MATERIALS: PART 4

Supplementary Methods

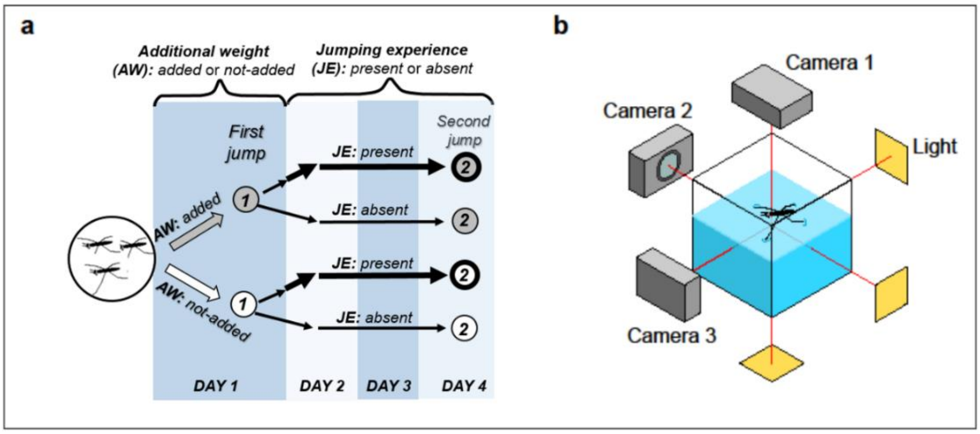

Supplementary Figure 2. Schematic illustration of the experimental design. (a) Water striders were divided in 4 groups (weight added + jumping experience present, weight added + jumping experience absent, weight not added + jumping experience present, weight not added + jumping experience absent). On the first day (DAY 1), the weights were added to the individuals in the weight added group. About 2 hours later the *First jump* was filmed (Results in Fig. 1). During the next three days (DAY 2 ~ DAY 4) *jumping experience present* groups experienced additional jumping, while *jumping experience absent* groups remained undisturbed. The *Second jumps* were recorded on DAY 4. (b) The arrangement of cameras and lights during filming.

Supplementary Table 7. Experimental treatment groups and sample sizes. Numbers indicate number of individuals for which both *First* and *Second* jumps were digitized and analyzed.

Additional weight (AW)	*Jumping experience (JE)*	**No. Females**	**No. Males**
weight-added	*present*	6	6
weight-added	*absent*	5	10
weight-not-added	*present*	12	8
weight-not-added	*absent*	8	7

Morphological measurements crucial for model calculations

Supplementary table 8. Body measurements of the males and females from the study population of *Gerris latiabdominis*: leg lengths and y_i of water striders used in this study. y_i, is the distance from body center to the undisturbed water surface in the resting position of the water strider. They were used in calculating the sex-specific critical threshold in Fig. 4.

		Length of mid legs (mm)			Length of hind legs (mm)			Length of y_i (mm)	
Sex	N	femur	tibia	tarsus	femur	tibia	tarsus	N	average
F	24	5.62 (0.81)	4.26 (0.53)	3.02 (0.15)	4.98 (0.18)	3.02 (0.32)	1.58 (0.18)	12 (6)	1.15 (0.40)
M	24	5.28 (0.39)	3.95 (0.38)	2.85 (0.31)	4.83 (0.39)	2.83 (0.24)	1.46 (0.17)	9 (5)	1.24 (0.40)

Supplementary References

1. Moran, D.M. Arguments for rejecting the sequential Bonferroni in ecological studies. *Oikos*. **100**, 403-405 (2003).
2. Nakagawa, S. A farewell to Bonferroni: the problems of low statistical power and publication bias. *Behavioral Ecology*. **15**, 1044-1045 (2004).
3. Rice, W.R. Analyzing tables of statistical tests. *Evolution*. **43**, 223-225 (1989).

SUPPLEMENTARY VIDEO - explanations

Baek et al Sci Rep Supplementary Video.mov
The file contains four jumps of *Gerris latiabdominis* extracted from four experimental movies by zooming in to include only the areas of interest and by showing side by side jumps without and with additional weight. These slow motion movies were shot at 500 fps and were further slowed down to the level of 70% of the typical 500 fps slow motion movie. Hence, if played at 30 fps (standard) the movies slow down reality by a factor of 0.042, i.e. we observe the jumps at ~4% of the real speed.

Baek et al Sci Rep Supplementary Video 2x slower with arrows.mov
This file contains the same video as above but slowed down 2x and red arrows are added to illustrate differences between weight-added and weight-not added individuals.
`
